# Simulating Electronic Coherences Induced by Conical Intersections Using MASH: Application to Attosecond X‑ray Spectroscopy

**DOI:** 10.1021/acs.jpclett.5c01407

**Published:** 2025-06-25

**Authors:** Daniele Furlanetto, Jeremy O. Richardson

**Affiliations:** Department of Chemistry and Applied Biosciences, 463172 ETH Zürich, 8093 Zürich, Switzerland

## Abstract

In this work, we employ trajectory-based simulations to predict the electronic coherences created by nonadiabatic dynamics near conical intersections. The mapping approach to surface hopping (MASH) is compared with standard fewest-switches surface hopping on three model systems, for which the full quantum-mechanical results are available. Electronic populations and coherences in the adiabatic representation, as well as nuclear densities, are computed to assess the robustness of the different methods. The results show that standard surface hopping can fail to describe the electronic coherences, whereas they are accurately captured by MASH at the same computational cost. In this way, MASH appears to be an excellent simulation approach for novel X-ray spectroscopies, such as the recently proposed transient redistribution of ultrafast electronic coherences in attosecond Raman signals (TRUECARS).

## Graphical abstract

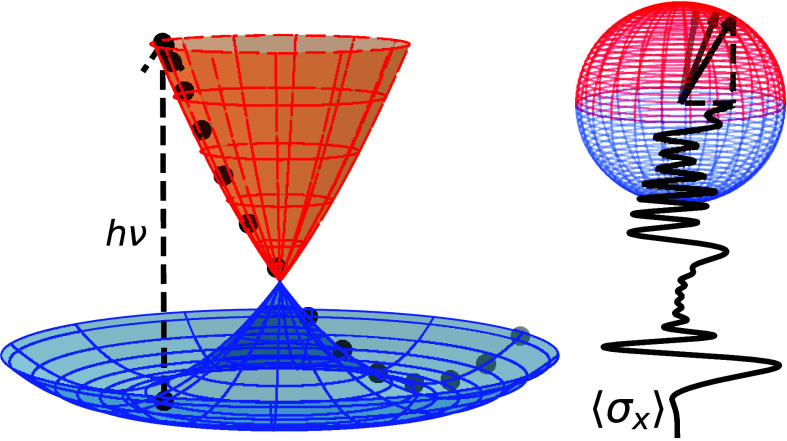

The nonadiabatic dynamics of photoexcited molecules is of fundamental interest because of its impact on chemical synthesis,^1,2^ biological processes,^3^ and medical advancement.^4^ Ultrafast internal energy conversion and other photochemical processes are typically mediated by conical intersections (CIs), where the ground- and excited-state potential energy surfaces are degenerate.^5^ Despite the fact that CIs are a theoretical construct, it has been proposed that there are physical signatures associated with them that could be measured experimentally.^6^ For instance, a novel X-ray spectroscopy called transient redistribution of ultrafast electronic coherences in attosecond Raman signals (TRUECARS) has been designed to probe the electronic coherences generated by the transition through a CI.^7^

Experimental spectroscopic signals are often interpreted using the help of theoretical simulations. Whereas it has become common to calculate the electronic population dynamics using semiclassical methods such as surface hopping,^8–10^ it is often assumed that coherences are much harder to capture than populations. One reason for this could be that the well-known overcoherence problem of FSSH is often tackled using decoherence corrections.^11–14^ However, these corrections artificially modify the wave-function coefficients, which could destroy the coherences that we wish to study. It is thus clearly preferable to avoid decoherence corrections in this case wherever possible. Therefore, (with the exception of one surface-hopping study)^15^ nearly all previous simulations of TRUECARS have relied on quantum-mechanical methods either in reduced dimensionality,^16^ for simplified system–bath models,^17^ or using trajectory-guided basis functions.^18^

The mapping approach to surface hopping (MASH)^19,20^ has recently emerged as an alternative to fewest-switches surface hopping (FSSH).^21,22^ In particular, it is deterministic rather than stochastic and thus avoids the problem of inconsistency between the active state and the electronic coefficients, which can plague FSSH.^11^ MASH is rigorously derived from a short-time limit of quantum mechanics^19,20^ and has been shown to fix known problems of FSSH with computing nonadiabatic rates^23^ or population observables starting from coherent states.^24^ Moreover, it maintains all the pros of an independent-trajectory method, allowing a first-principles on-the-fly simulation of complex molecular systems in full dimensionality.^25,26^ In fact, MASH can be implemented with an algorithm based on wave-function overlaps similar to that of FSSH, although with the added benefit of being reversible and second-order in time.^27^

In order to assess the accuracy of the MASH and FSSH methods for the evaluation of coherences, we will test them on various model systems, which describe both avoided crossings and CIs, and compare the results with fully quantum-mechanical (QM) calculations. In this way, we find MASH to be a powerful method for simulating the coherences generated in nonadiabatic processes and propose that it can be used to reliably predict TRUECARS signals in molecular systems.

In this work, we focus on electronic coherences in two-state systems and describe how to calculate them with both quantum-mechanical and semiclassical methods. Our formalism will be based on Pauli matrices defined in the adiabatic representation:^20^

1a $${\mathrm{\sigma ̂}}_{x}=\left|\phi_{0}\right\rangle \left\langle \phi_{1}\right|+\left|\phi_{1}\right\rangle \left\langle \phi_{0}\right|$$

1b $${\mathrm{\sigma ̂}}_{y}=i\left(\left|\phi_{0}\right\rangle \left\langle \phi_{1}\right|-\left|\phi_{1}\right\rangle \left\langle \phi_{0}\right|\right)$$

1c $${\mathrm{\sigma ̂}}_{z}=\left|\phi_{1}\right\rangle \left\langle \phi_{1}\right|-\left|\phi_{0}\right\rangle \left\langle \phi_{0}\right|$$

where |ϕ_0_⟩ and |ϕ_1_⟩ are the ground and excited electronic states, respectively. The Pauli matrices are thus implicitly dependent on the position of the nuclei, *q*. We chose to work in the adiabatic representation because this is the natural choice for FSSH and MASH. However, note that the TRUECARS signal, which we will evaluate below, is, like all physical observables, formally independent of the representation used.

In quantum mechanics, the total (nuclear and electronic) wave function can be expanded in the adiabatic basis as |Ψ­(*t*)⟩ = |χ_0_(*t*)⟩ ⊗ |ϕ_0_⟩ + |χ_1_(*t*)⟩ ⊗ |ϕ_1_⟩, although in practice we propagate it using the split-operator method in the diabatic basis and only convert to the adiabatic representation before evaluating observables. The quantum-mechanical definitions of the coherences are ⟨σ_*x*_(*t*)⟩ = 2Re⟨χ_1_(*t*)|χ_0_(*t*)⟩ and ⟨σ_*y*_(*t*)⟩ = 2Im⟨χ_1_(*t*)|χ_0_(*t*)⟩, whereas the population difference is given by ⟨σ_*z*_(*t*)⟩ = ⟨χ_1_(*t*)|χ_1_(*t*)⟩ – ⟨χ_0_(*t*)|χ_0_(*t*)⟩. It is clear that coherences require measurement of the overlap of two different wavepackets, whereas the population difference is defined purely in terms of the probabilities of the adiabatic states. For this reason, it is often assumed that the populations are easier to model with semiclassical methods. However, coherences carry phase information, which can be very important for novel spectroscopies, such as TRUECARS.

In FSSH, each trajectory corresponds to an electronic wave function, defined as |ψ­(*t*)⟩ = *c*_0_(*t*)|ϕ_0_⟩ + *c*_1_(*t*)|ϕ_1_⟩, while the active surface, *n* ∈ {0, 1}, is an additional time-dependent stochastic variable that determines the force used to evolve the nuclei. There have been multiple suggestions for how to measure electronic observables in FSSH; we follow the procedure of using the wave-function coefficients for the coherences and active state *n* to measure the populations:^22^ ⟨σ_*x*_(*t*)⟩ = ⟨2Re­[*c*_0_(*t*)*c*_1_^*^(*t*)]⟩_FSSH_, ⟨σ_*y*_(*t*)⟩ = ⟨2Im­[*c*_0_(*t*)*c*_1_^*^(*t*)]⟩_FSSH_, and ⟨σ_*z*_(*t*)⟩ = ⟨δ_1,*n*(*t*)_ – δ_0,*n*(*t*)_⟩_FSSH_. Here, the FSSH average is taken over an ensemble of trajectories initialized with nuclear phase-space variables sampled randomly from a Wigner distribution and where the electronic wave function of each trajectory is initialized in the excited state (|ψ(0)⟩ = |ϕ_1_⟩).

In MASH, the role of the electronic wave function is taken by a spin vector that evolves on a Bloch sphere. The nuclear force is determined by the *S*_*z*_ component of the spin. When it is in the lower/upper hemisphere, the ground/excited-state force is used. The expectation values are written as correlation functions:

2a $$\left\langle \sigma_{x}\left(t\right)\right\rangle ={\left\langle 2h\left(S_{z}\left(0\right)\right)S_{x}\left(t\right)\right\rangle }_{\mathrm{MASH}}$$

2b $$\left\langle \sigma_{y}\left(t\right)\right\rangle ={\left\langle 2h\left(S_{z}\left(0\right)\right)S_{y}\left(t\right)\right\rangle }_{\mathrm{MASH}}$$

2c $$\left\langle \sigma_{z}\left(t\right)\right\rangle ={\left\langle 2\left|S_{z}\left(0\right)\right|h\left(S_{z}\left(0\right)\right)\;\mathrm{sgn}\left(S_{z}\left(t\right)\right)\right\rangle }_{\mathrm{MASH}}$$

where *h* is the Heaviside step function and the average is taken over random samples of nuclear phase-space variables from a Wigner distribution with spin vectors uniformly distributed over the Bloch sphere.[Fn fn1] As we are initializing in a population, weighting functions of 2 or 2|*S*_*z*_(0)| are introduced according to the MASH prescription.^19,20^

In both our MASH and FSSH simulations, momenta are rescaled along the direction of the nonadiabatic coupling vectors after successful hops and are reflected for all frustrated hops. This procedure is uniquely derived from the MASH formalism,^19^ while for FSSH, numerous alternatives have been suggested.^28–30^

To highlight the differences between FSSH and MASH predictions of coherences, we utilize a system of coupled one-dimensional harmonic oscillators exhibiting an avoided crossing, which is described by the following diabatic potential (whose eigenvectors are the adiabatic states):

3 $$\mathrm{V̂}=\frac{1}{2}m\omega^{2}q^{2}+\left(\begin{array}{cc} \kappa q+\varepsilon & \Delta \\ \Delta & -\kappa q-\varepsilon \end{array}\right)$$

with the parameters defined in the Supporting Information.

Figure 1 shows that both semiclassical methods correctly describe the population evolution, despite the fact that FSSH exhibits a large inconsistency error, as seen by the discrepancy between the average populations measured by the wave-function coefficients and the fraction of trajectories on the given state. The nuclear-density evolution also shows comparable results for all methods (Figure 1). The only significant difference is that the full QM simulation shows signs of nuclear interference near *t* = 5. This is the only feature of the simulations that cannot be captured by MASH. However, it does not affect the quality of the electronic coherences, which are in excellent agreement at all times.

**Figure 1:**
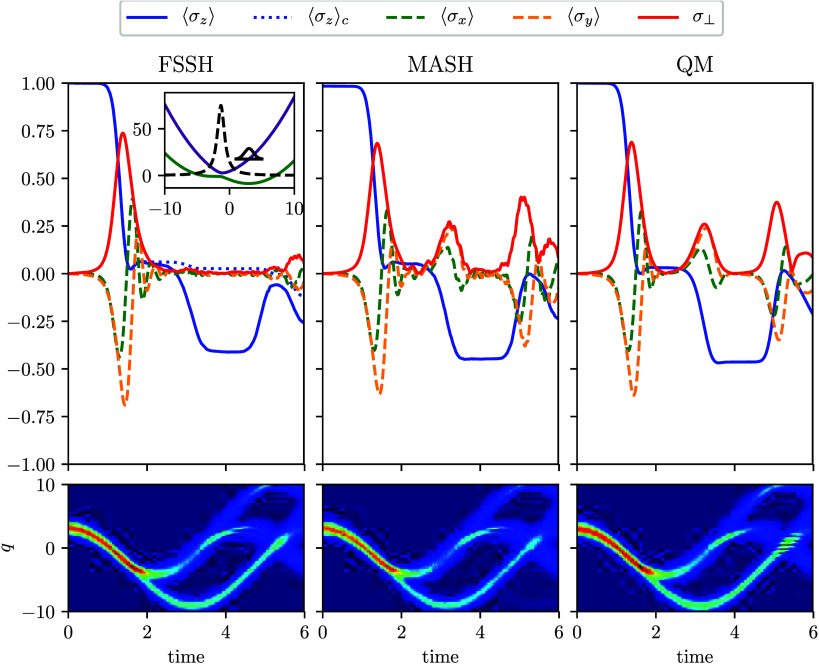
Adiabatic populations and coherences calculated by semiclassical and quantum methods for the avoided-crossing model (top row). The alternative FSSH measure of ⟨σ_*z*_(*t*)⟩_c_ = ⟨|*c*_1_(*t*)|^2^ – |*c*_0_(*t*)|^2^⟩_FSSH_ is also shown to illustrate the inconsistency with ⟨σ_*z*_(*t*)⟩. $\sigma_{⊥}=\sqrt{\langle \sigma_{x}\rangle^{2}+\langle \sigma_{y}\rangle^{2}}$ is the absolute value of the coherences. The inset shows the adiabatic energies, nonadiabatic coupling, and initial wavepacket. Time-dependent nuclear density (bottom row).

In contrast, FSSH fails completely to describe the coherences induced by the second and third passages through the coupling region (*t* ≈ 3 and *t* ≈ 5). This is a direct consequence of the inconsistency problem in FSSH, which is fixed in MASH. This is a clear numerical example of the benefits of the rigorous MASH prescription over the traditional FSSH approach. Similar behavior was observed for Tully’s third model in ref 19.

In two or more dimensions, it is possible (and quite common) to encounter conical intersections. CIs are known to play a key role in photochemical reactions,^5^ and it is therefore of great interest to study the dynamics in their vicinity. For this work, we study the coherences generated on passing through a conical intersection. One might assume that due to the huge nonadiabatic coupling in this region, the coherence signals will be large. However, if the nuclear wavepacket is spread on both sides of the CI, the paths that pass by the CI on different sides will experience opposite nonadiabatic couplings, leading to total destructive interference of the coherences.^31–33^

To illustrate this behavior, we used a simple symmetric Jahn–Teller model described by the following diabatic potential:

4 $$\mathrm{V̂}=\frac{1}{2}m\omega^{2}\left({q_{1}}^{2}+{q_{2}}^{2}\right)+\left(\begin{array}{cc} \kappa q_{1} & \lambda q_{2}\\ \lambda q_{2} & -\kappa q_{1} \end{array}\right)$$

Solving for the adiabatic energies (as the eigenvalues of this matrix) leads to a peak CI at the origin. The initial wavepacket is chosen to be a Gaussian on the excited state, centered at a positive *q*_1_ with zero average momentum; the initial conditions are thus symmetric with respect to reflection in the *q*_2_ axis. As the symmetric wavepacket passes near the CI, it generates an antisymmetric wavepacket on the ground state due to antisymmetric coupling *d*·*p*, where $d=\left\langle \phi_{1}\right|\frac{\partial }{\partial q}\phi_{0}\rangle$ is the nonadiabatic coupling and *p* is the momentum. Coherences ⟨σ_*x*_⟩ and ⟨σ_*y*_⟩ will thus be zero since overlap ⟨χ_1_(*t*)|χ_0_(*t*)⟩ vanishes due to the different symmetries of the wave functions.[Fn fn2]

The MASH coherences are also zero by symmetry. For every trajectory starting with the initial conditions (*q*_1_, *q*_2_, *p*_1_, *p*_2_, *S*_*x*_, *S*_*y*_, *S*_*z*_), there is another trajectory with the same weight starting with (*q*_1_, −*q*_2_, *p*_1_, −*p*_2_, −*S*_*x*_, −*S*_*y*_, *S*_*z*_). These two trajectories will experience equivalent forces but opposite coupling *d*·*p*, leading to antisymmetric evolution of the spin vector and therefore zero electronic coherences on average. For a similar reason, fully converged FSSH simulations also give zero coherences.

The numerical results in Figure 2 confirm that as expected the coherences are zero throughout the entire dynamics. Nonetheless, the populations show that nonadiabatic transitions are taking place. The FSSH population is clearly affected by inconsistency with the electronic coefficients and leads to poor predictions after the third crossing region, whereas MASH is significantly more accurate.

**Figure 2:**
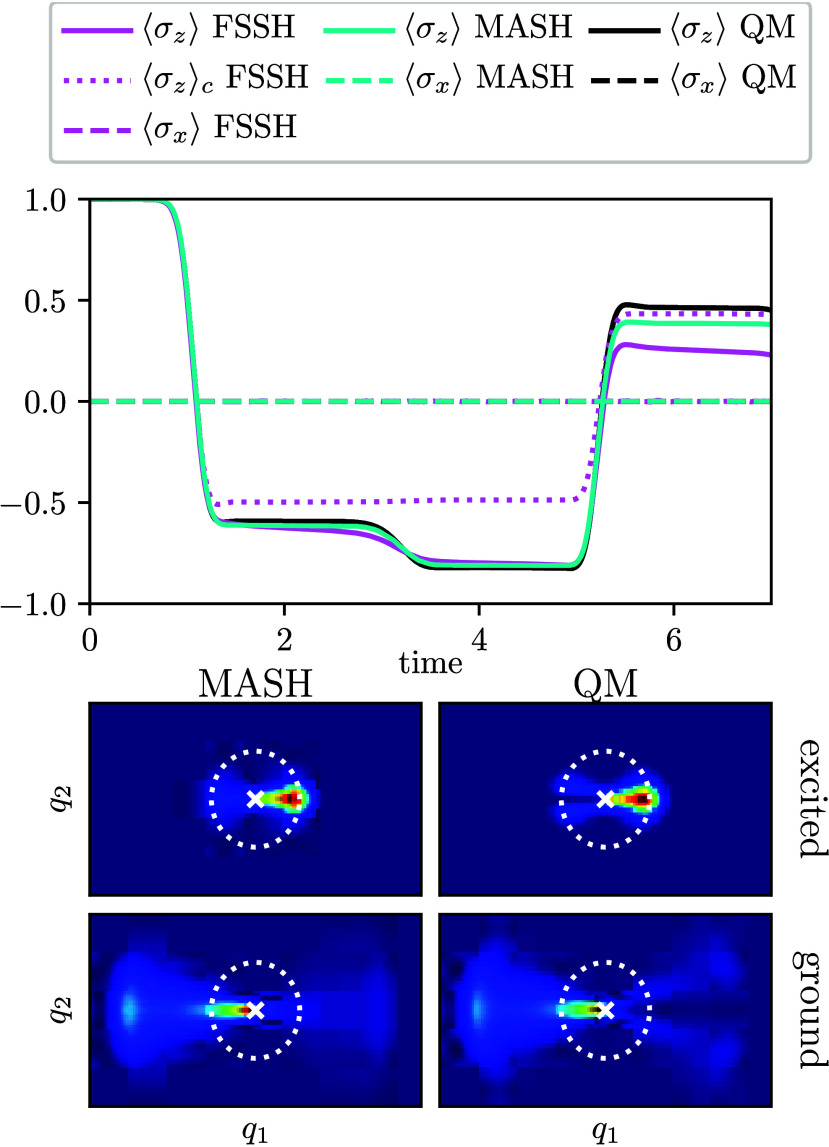
Time-dependent populations and coherences for the Jahn–Teller model with symmetric initial conditions (top). Note that the coherences are zero in all methods. MASH and QM nuclear densities on the excited and ground states averaged over the simulation time (bottom). The white dotted circle indicates the degenerate ground-state minimum, and the white cross indicates the CI. A node in the QM density is visible on the right (left) of the CI for the ground (excited) state.

The geometric-phase effect^34^ influences the nuclear wave function by enforcing a node around the CI. This effect can be observed in the quantum nuclear density plotted in Figure 2.^35,36^ In MASH and FSSH, the nuclear densities do not exhibit a node because they do not capture nuclear interference. It is thus clear that the suppression of coherences is caused by the symmetry of the problem^32^ and is not directly a geometric-phase effect as has been suggested in the literature.^31^

Starting the wavepacket with a nonzero average initial momentum in the direction of *q*_2_ breaks the symmetry. The wavepacket orbits around the CI, resulting in a dynamics that can be interpreted as multiple passages through avoided-crossing regions. The nuclear density (Figure 3) evolves similarly for all methods. The electron population dynamics are best captured by MASH, whereas FSSH is again strongly affected by internal inconsistency. The electron coherence dynamics is captured by all methods in the first and second crossings, but FSSH fails completely in the third crossing (*t* ≈ 5.5). There is no obvious effect of the geometric phase here since the whole wavepacket goes around the CI on one side, such that there is no nuclear interference.

**Figure 3:**
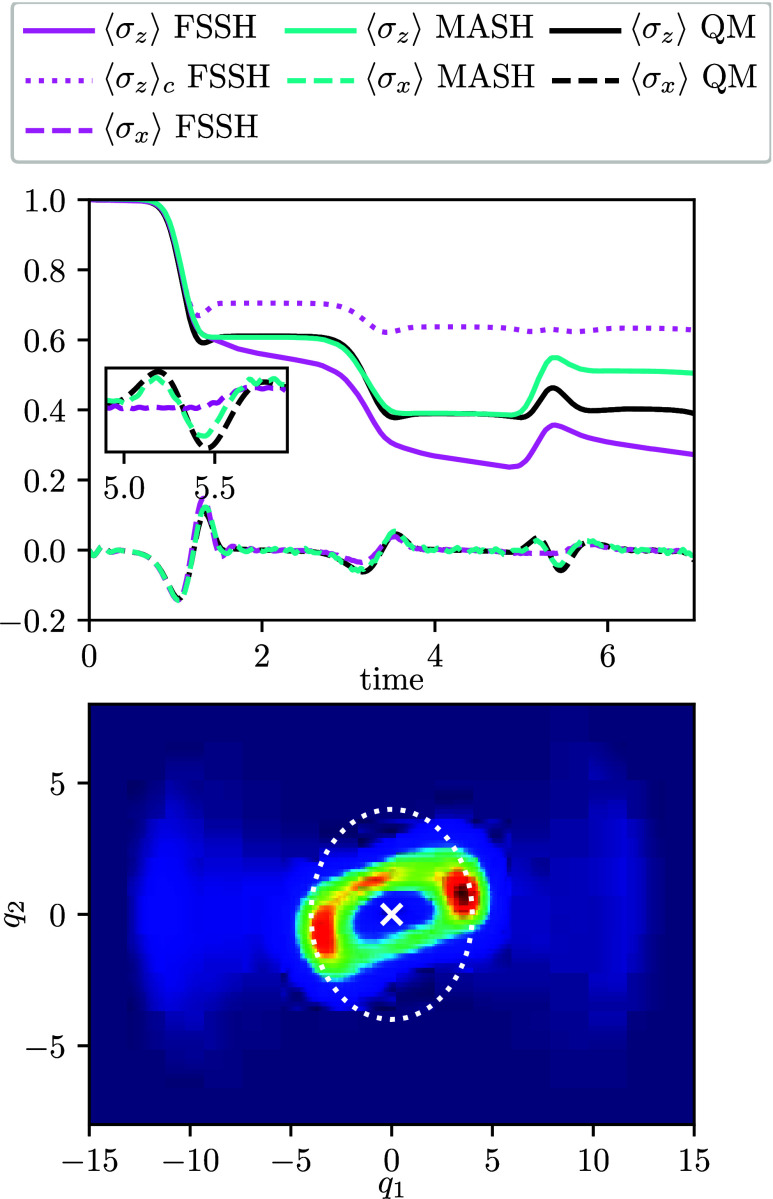
Populations and coherences for the Jahn–Teller model with asymmetric initial conditions (top). The inset shows the coherences around the third crossing (*t* ≈ 5.5). QM nuclear density averaged over the simulation time (bottom). MASH and FSSH give almost identical results (see the Supporting Information).

The TRUECARS experiment was proposed to study the passage of a wavepacket through a region of nonadiabatic coupling, such as near a CI.^7^ The experimental observable is the intensity of the scattered Raman signal of a hybrid X-ray probe pulse after an optical pump has initialized the system in an excited-state population. The relevant response function^37^ is the time-dependent expectation value of polarizability ⟨α­(*t*)⟩. The spectroscopic signal can then be simulated as a function of time delay *T* and Raman shift ω as^7^

5 S(ω,T)=2Im⁡∫−∞+∞dteiω(t−T)EB*(ω)EN(t−T)⟨α(t)⟩

where $E_{B}\left(\omega \right)$ and $E_{N}\left(t\right)$ are the broadband and narrowband probe–pulse envelopes, respectively. Finally, an alternative way of visualizing the results is offered by the Wigner spectrogram:^16^

6 $$W\left(T,\omega \right)=\int_{-\infty }^{+\infty }dtS\left(\omega_{R},T+\frac{t}{2}\right)S\left(\omega_{R},T-\frac{t}{2}\right)e^{-i\omega t}$$

which is defined using a fixed value for frequency ω_R_.

The computations of S and W are just simple postprocessing operations. All that is required is expectation value ⟨α­(*t*)⟩, which can be computed by QM, MASH, or FSSH simulations. For instance, in quantum mechanics, ⟨α­(*t*)⟩ = ⟨Ψ­(*t*)|α̂|Ψ­(*t*)⟩. FSSH and MASH are formulated in the adiabatic basis, and thus, it is useful to decompose the real-value polarizability operator into the basis of adiabatic Pauli matrices as

7 $$\mathrm{\alpha ̂}\left(q\right)=\alpha_{z}\left(q\right){\mathrm{\sigma ̂}}_{z}+\alpha_{x}\left(q\right){\mathrm{\sigma ̂}}_{x}$$

In general, the polarizability operator is a tensor in the spatial coordinates. Here, we focus on the scalar isotropic component, although the generalization is trivial. Using eq 7, the FSSH definition of adiabatic populations and coherences, and the MASH coherences (eq 2a), we obtain the expectation values:

8a ⟨α(t)⟩=⟨αz(q(t))(δ1,n(t)−δ0,n(t))+2αx(q(t))Re[c1*(t)c0(t)]⟩FSSH

8b $$\left\langle \alpha \left(t\right)\right\rangle ={\left\langle 2\left|S_{z}\left(0\right)\right|h\left(S_{z}\left(0\right)\right)\alpha_{z}\left(q\left(t\right)\right)\;\mathrm{sgn}\left(S_{z}\left(t\right)\right)+2h\left(S_{z}\left(0\right)\right)\alpha_{x}\left(q\left(t\right)\right)S_{x}\left(t\right)\right\rangle }_{\mathrm{MASH}}$$

The polarizability thus has contributions from both the populations and the coherences, although the coherences are expected to dominate the TRUECARS signal as the Fourier transform of eq 5 will pick out oscillating terms.

To test the reliability of semiclassical methods for simulating TRUECARS observables, we used the nonlinear two-dimensional parametrized model of acrolein from ref 7, with minor modifications to ensure that the potential is bounded (see the Supporting Information). As in the original work, the dynamics are initialized in the excited adiabatic state with a Gaussian wavepacket with zero average momentum.

Figure 4 shows the time evolution of the polarizability for which the MASH result closely resembles the QM result. However, FSSH makes significant errors. In particular, the amplitudes of the oscillations are underestimated after about *t* ≈ 1200. This is a consequence of its poor description of the coherences (see the Supporting Information).

**Figure 4:**
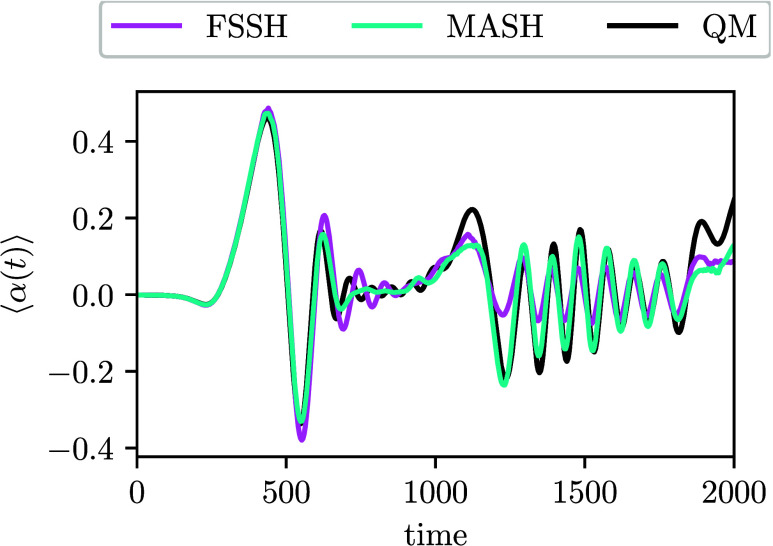
Time evolution of the polarizability expectation value for the model system of acrolein.

Postprocessing the time-dependent polarizability from each method gives the TRUECARS signals, S(ω,T) , shown in Figure 5. MASH gives an accurate prediction, whereas FSSH shows much weaker coherence at longer times. The error in FSSH is also clearly highlighted by the Wigner spectrogram, where the signal is almost completely washed out.

**Figure 5:**
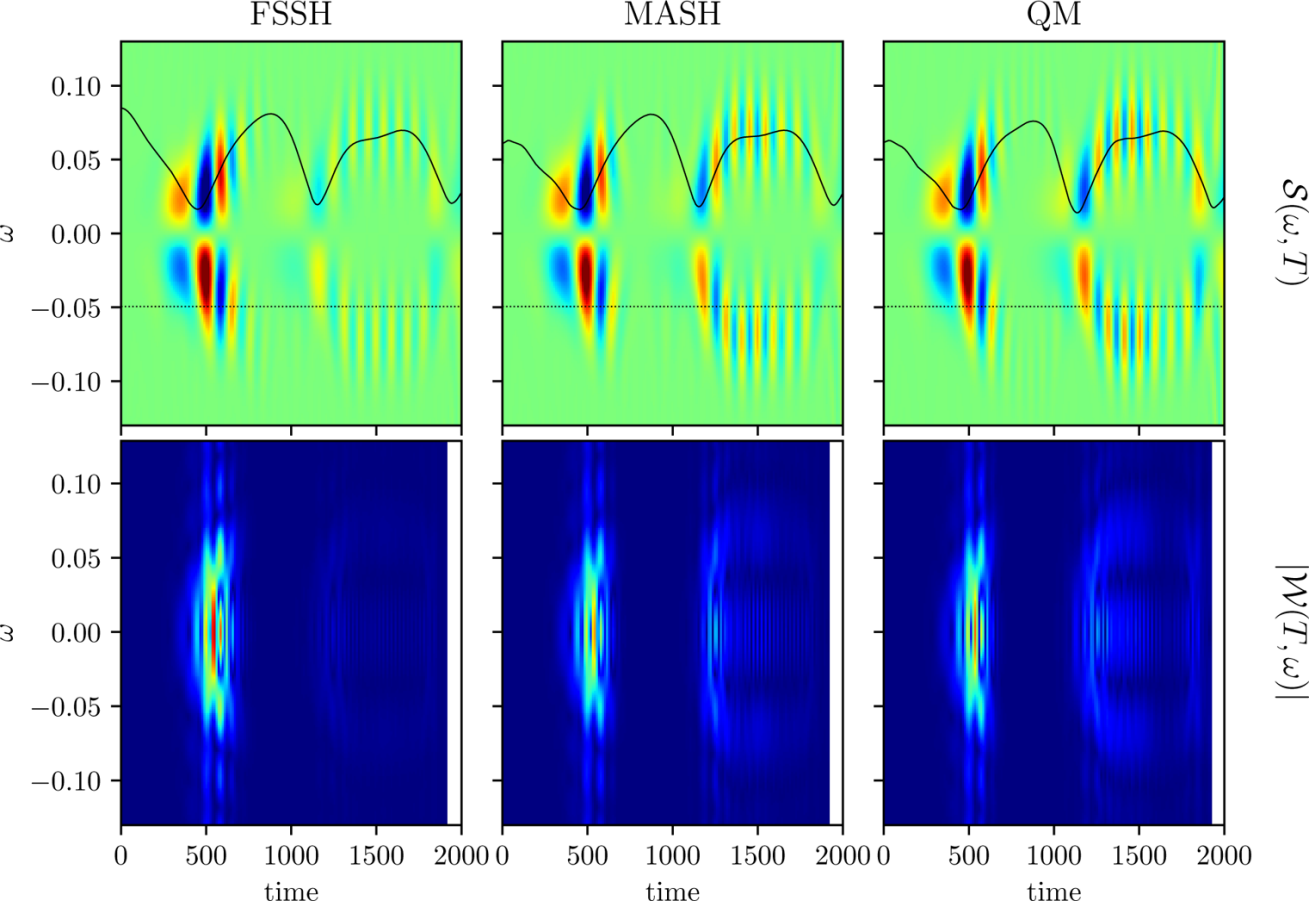
Spectroscopic signals S(ω,T) overlaid with ω̅(*T*) (top). Absolute values of Wigner spectrograms |W(T,ω)| for reference frequency ω_R_ (bottom) shown by a dotted line in the upper panels. All quantities are given in atomic units.

In addition to predicting the TRUECARS signal, we can use the ensemble of MASH trajectories to interpret it. In order to do this, we adapt an idea from ref 7 to relate the envelope of the signal to time-dependent adiabatic energy gaps *ΔV*(*q*) = *V*_1_(*q*) – *V*_0_(*q*). A justification for this connection is presented in the Supporting Information.

In Figure 6, we present a time-dependent histogram of ω­(*t*) = *ΔV*(*q*(*t*)) from the ensemble of MASH trajectories weighted by $2h\left(S_{z}\left(0\right)\right)\sqrt{{S_{x}}^{2}\left(t\right)+{S_{y}}^{2}\left(t\right)}$ , whose shape roughly follows that of the envelope of S(ω,T) . For comparison, we also plot the mean frequency of this distribution, ω̅(*t*), on both Figures 5 and 6. It is clear from the minima in ω̅(*t*) that the trajectories pass through a region of strong nonadiabatic coupling (where the energy gap is small) three times. Moreover, after the second crossing, a large proportion of the trajectories find themselves in regions with similar potential-energy gaps (*ΔV* ≈ 0.07). The spin vectors of this set of trajectories thus evolve in phase and are what leads to the long-lasting coherences seen in the polarizability.

**Figure 6:**
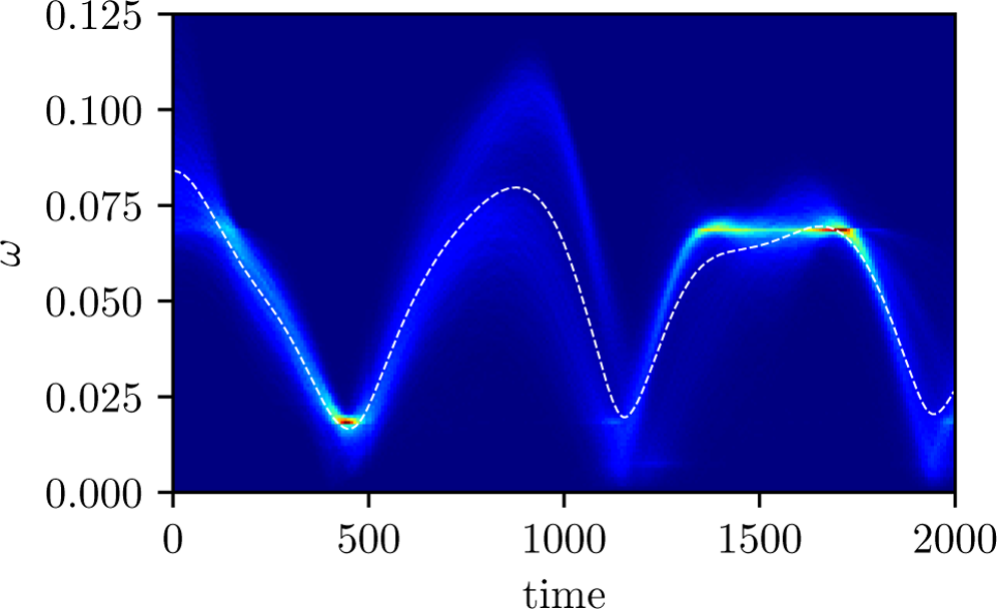
Energy shift between adiabatic states *ΔV*, measured from the ensemble of MASH trajectories weighted by corresponding coherence factor $\sqrt{S_{x}{\left(t\right)}^{2}+S_{y}{\left(t\right)}^{2}}$ . Time-dependent mean frequency ω̅(*t*) is overlaid as a dashed line.

The Wigner spectrogram shows a strong feature at *T* ≈ 500 corresponding to the creation of coherence during the first nonadiabatic transition. Its spread in the range ω ≈ [−0.06, 0.06] represents the potential-energy splitting in this region and includes a significant contribution from the conical intersection degeneracy at ω = 0. At longer times *T* ≳ 1200, we observe three horizontal stripes in the Wigner spectrogram at ω ≈ −0.07, 0, and 0.07. Reference 16 interpreted similar features in terms of the dominating vibronic eigenstates that contribute to the coherence and claimed that a classical treatment could not describe such time-independent quantum states. However, our MASH simulation seems to capture the behavior almost perfectly, despite being based on an ensemble of classical trajectories with time-dependent energy splittings. This implies that the horizontal features may not be signatures of quantum-mechanical vibronic eigenstates at all but rather just electronic coherences from different parts of the trajectory ensemble.

In conclusion, our results show that it is possible to accurately calculate observables based on electronic coherences using semiclassical MASH dynamics, despite the fact that nuclear quantum effects are neglected. In contrast, the FSSH method fails not because of the semiclassical approximation per se but because of its well-known overcoherence problem leading to its inconsistency error. Even in cases where FSSH gives reasonable predictions of the populations, it can fail dramatically for the coherences. This is related to the fact that more reliable population results are obtained from active surfaces than from electronic coefficients, but that the coherences have to be computed from electronic coefficients. MASH, on the other hand, does not suffer from the inconsistency error and can predict accurate populations and coherences from its ensemble of classical trajectories.

Although we have focused on two-state systems here, MASH has been recently generalized to multistate systems. For photochemistry, we recommend the approach of ref 38, whereas for exciton models, refs 39 and 40 are preferred.^20^ Additionally, as the MASH algorithm has the same computational cost as FSSH, it can also be used with on-the-fly electronic-structure calculations.^25,26,41^ This will allow reliable ab initio simulations of coherences in molecules treated in full dimensionality. In this way, we can provide mechanistic understanding and theoretical support to novel nonlinear spectroscopies such as TRUECARS.

## Supplementary Material





## Footnotes

a Due to factor *h*(*S*_*z*_(0)), only trajectories initialized in the northern hemisphere will contribute, and thus, the sampling can be limited to that region.

b Note that the diabatic coherences are also zero by similar symmetry arguments.

## Notes

System parameters, initial conditions for the simulations and the definition of the postprocessing procedure, additional results of the time-dependent polarizability and coherences, and symmetry treatment of the Jahn–Teller model (PDF)
Transparent Peer Review report available (PDF)
