# Supplementary material for: Simulating Electronic Coherences Induced by Conical Intersections Using MASH: Application to Attosecond X‑ray Spectroscopy
Source: J Phys Chem Lett. 2025 Jun 25;16(26):6794–800. doi: 10.1021/acs.jpclett.5c01407 (PMC12235616; doi:10.1021/acs.jpclett.5c01407)
Supplement: Supplementary file 1 [file jz5c01407_si_001.pdf]

Supporting Information
Simulating electronic coherences induced by conical intersections using MASH: Application to attosecond X-ray spectroscopy

Daniele Furlanetto* and Jeremy O. Richardson*

Department of Chemistry and Applied Biosciences, ETH Zürich, 8093 Zürich, Switzerland

E-mail: daniele.furlanetto@phys.chem.ethz.ch; jeremy.richardson@phys.chem.ethz.ch

Time-dependent polarizability We only need to treat the traceless part of the polarizability operator $\hat{\alpha}$, since an identity component of this operator gives zero signal in TRUECARS. Note also that the polarizability is real as the Hamiltonian is real. We will additionally choose the diagonal contributions to the diabatic polarizability to be zero, as suggested in Ref. 7, as they only contribute to the signal during the nonadiabatic passage and not to the long-lived coherences. Moreover we choose the diabatic polarizability to be independent of nuclear position and note that the proportionality constant is unimportant. Thus, in the *diabatic* representation, the polarizability operator is

$$\hat{\alpha}(q) = \begin{pmatrix} 0 & 1 \\ 1 & 0 \end{pmatrix} \tag{1}$$

for all our models.

The *adiabatic* components of the polarizability can be obtained using the adiabatic Pauli matrices (which are implicitly q-dependent):

$$\alpha_x(q) = \tfrac{1}{2}\,\mathrm{Tr}[\hat{\alpha}(q)\hat{\sigma}_x] \tag{2a}$$

$$\alpha_z(q) = \tfrac{1}{2}\,\mathrm{Tr}[\hat{\alpha}(q)\hat{\sigma}_z] \tag{2b}$$

Note that the $\alpha_y(q)$ component of the polarizability is zero since we assume the operator $\hat{\alpha}(q)$ to be real. Note that even if the diabatic polarizability is constant, the adiabatic polarizability will depend on the nuclear position through the Pauli matrices. The polarizability of real molecules would have tensorial spatial parts; each component can be treated in exactly the same way as above.

Avoided-crossing model The avoided-crossing model described in Eq. (3) of the main paper is defined using the following parameters in reduced units: $\hbar = 1$, $m = 1$, $\omega = 1$, $\kappa = 3$, $\varepsilon = 4$, $\Delta = 2$.

The initial wavefunction on the excited adiabatic state is described by a Gaussian wavepacket (centred at $q = \kappa/(m\omega^2)$ and $p = 0$ with a standard deviation that corresponds to ω). The semiclassical trajectories are sampled from the corresponding Wigner distribution.

$$\chi_1(q, t = 0) = \sqrt[4]{\frac{m\omega}{\pi\hbar}}\, \mathrm{e}^{-\frac{m\omega}{2\hbar}(q - \frac{\kappa}{m\omega^2})^2} \tag{3a}$$

$$\rho_1(q, p, t = 0) = \frac{1}{\pi\hbar}\, \mathrm{e}^{-\frac{m\omega}{\hbar}(q - \frac{\kappa}{m\omega^2})^2}\, \mathrm{e}^{-\frac{p^2}{m\hbar\omega}} \tag{3b}$$

These initial conditions model an instantaneous excitation at the Franck–Condon point.

Throughout the entire paper, the nuclear dynamics are integrated using the velocity-Verlet algorithm, while the electronic dynamics are integrated using the nonadiabatic coupling. For MASH the electronic integration is symmetrized and bisected 10 times in the jumping steps.[27] The simulation timestep is $\delta t = 0.02$, which ensures convergence of the trajectory integration. For MASH and FSSH the convergence of the ensemble has been ensured using 10^5 trajectories. However, in practice it is not necessary to use so many. In Figure S1 the results are compared for different numbers of trajectories, showing that MASH and FSSH converge at a similar rate and that in both cases, reasonable results can already be obtained using about 1000 trajectories. Further analysis is provided by Figure S2, in which the estimated standard errors of MASH and FSSH are presented for both populations and coherences. Both MASH and FSSH numerical results obey the central-limit theorem, implying that the error bars scale with $1/\sqrt{N_{\mathrm{traj}}}$, where N_{traj} is the number of trajectories. However, there are subtle differences in the prefactor for this convergence behaviour. The population convergence is similar for MASH and FSSH, while the errors in the coherences

show a factor of about 2 in favour of FSSH. Note that it might be possible to improve the MASH convergence by developing a specialized importance sampling scheme. Most importantly, however, is that MASH gives statistically significant information on the correct behaviour of the coherences with between 100 and 1000 trajectories, whereas FSSH suffers from a systematic error.

To facilitate the convergence of the coherences at short times, the MASH spin-vector is sampled in a symmetric manner with respect to rotations about the z-axis. In practice for every trajectory with initial conditions (q, p, S_x, S_y, S_z), another trajectory is initialized with $(q, p, -S_x, -S_y, S_z)$. This approach speeds up the convergence of the results (at least at short times).

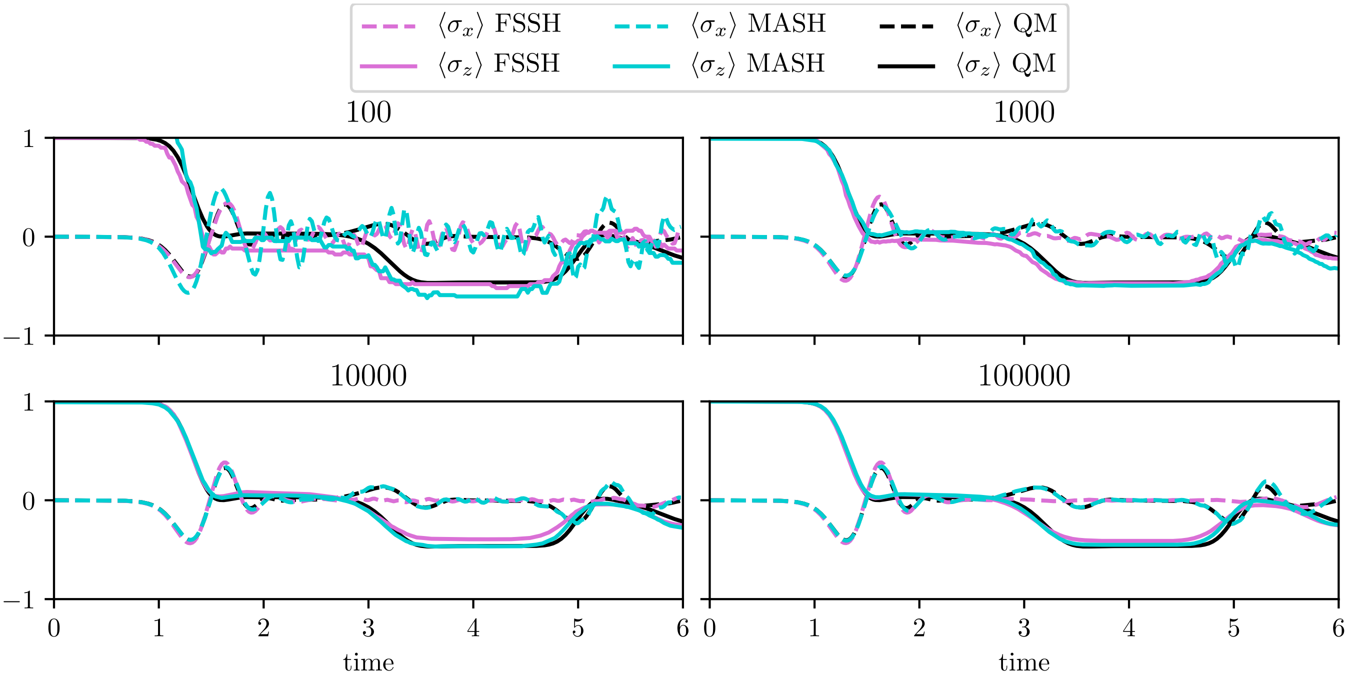

Figure S1: MASH and FSSH populations and coherences averaged over different numbers of trajectories for the avoided-crossing model.

Figure S3 shows the time-dependent expectation value of the polarizability obtained from the simulations described in the main text. As a consequence of its error in computing adiabatic coherences, the FSSH polarizability shows significant differences from the full quantum-mechanical (QM) results, especially near the peaks at $t \approx 3.5$, 5 and 5.3. In contrast, the MASH prediction is in much better agreement (although the peak at $t \approx 5$ still shows a small discrepancy).

2D Jahn–Teller models The symmetric Jahn–Teller model described in Eq. (4) of the main text is defined using the following parameters: $\hbar = 1$, $m = 1$, $\omega = 1$, $\kappa = \lambda = 4$ in reduced units. The initial wavefunction is described by a Gaussian wavepacket in the excited adiabatic state which is centered in position $\bar{q} = (\kappa/m\omega^2, 0)$ with a standard deviation that corresponds to ω in both directions. The average initial momentum $\bar{p} = (0, 0)$ in the first simulation, while it is $\bar{p} = (0, 2)$ in the second case. Notice that non-symmetric initial conditions can be obtained setting $\kappa \neq \lambda$ and shifting the initial wavepacket. In both cases, the semiclassical trajectories are sampled from the corresponding Wigner distributions.

$$\chi_1(q, t = 0) = \sqrt{\frac{m\omega}{\pi\hbar}}\, \mathrm{e}^{-\frac{m\omega}{2\hbar}(q_1-\bar{q}_1)^2}\, \mathrm{e}^{-\frac{m\omega}{2\hbar}q_2^2+\frac{\mathrm{i}}{\hbar}\bar{p}_2 q_2} \tag{4a}$$

$$\rho_1(q_1, q_2, p_1, p_2, t = 0) = \frac{1}{\pi^2\hbar^2}\, \mathrm{e}^{-\frac{m\omega}{\hbar}(q_1-\bar{q}_1)^2}\, \mathrm{e}^{-\frac{m\omega}{\hbar}q_2^2}\, \mathrm{e}^{-\frac{p_1^2}{m\hbar\omega}}\, \mathrm{e}^{-\frac{(p_2-\bar{p}_2)^2}{m\hbar\omega}} \tag{4b}$$

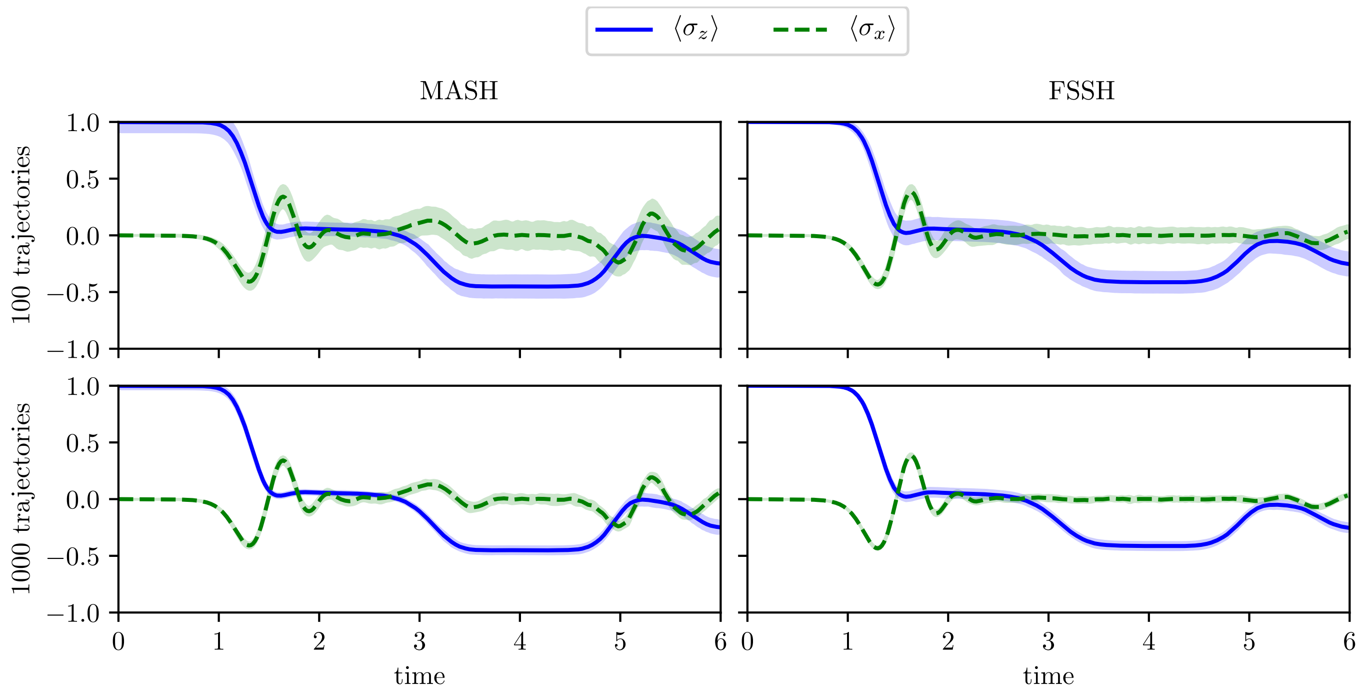

Figure S2: Estimates of the standard error for MASH and FSSH populations and coherences given ensembles of different numbers of trajectories for the avoided-crossing model.

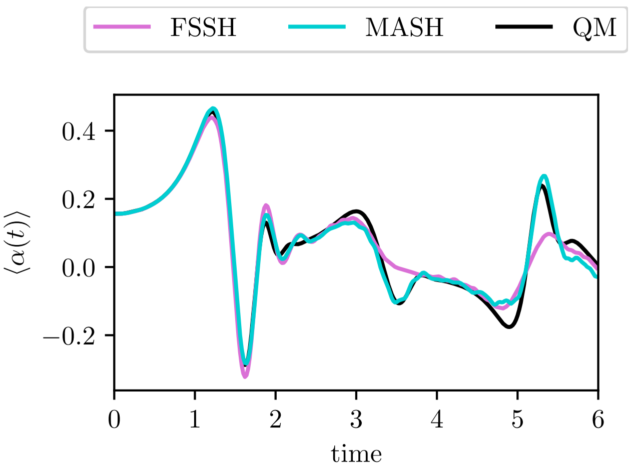

Figure S3: Time-evolution of the polarizability expectation value for the avoided-crossing model.

The simulation timestep is $\delta t = 0.01$. For MASH and FSSH the convergence has been ensured using 10^5 trajectories. In the case of the first simulation, the trajectory sampling is symmetrized in the nuclear phase-space for both MASH and FSSH: for each trajectory with initial conditions $(q_1, q_2, p_1, p_2, S_x, S_y, S_z)$, another trajectory is initialized with $(q_1, -q_2, p_1, -p_2, -S_x, -S_y, S_z)$. Using this symmetrized approach, the MASH coherences are exactly zero at each time. The FSSH coherences are not ensured to be zero (except in the limit of infinite sampling), since the random numbers for the hopping algorithm are not symmetrized.

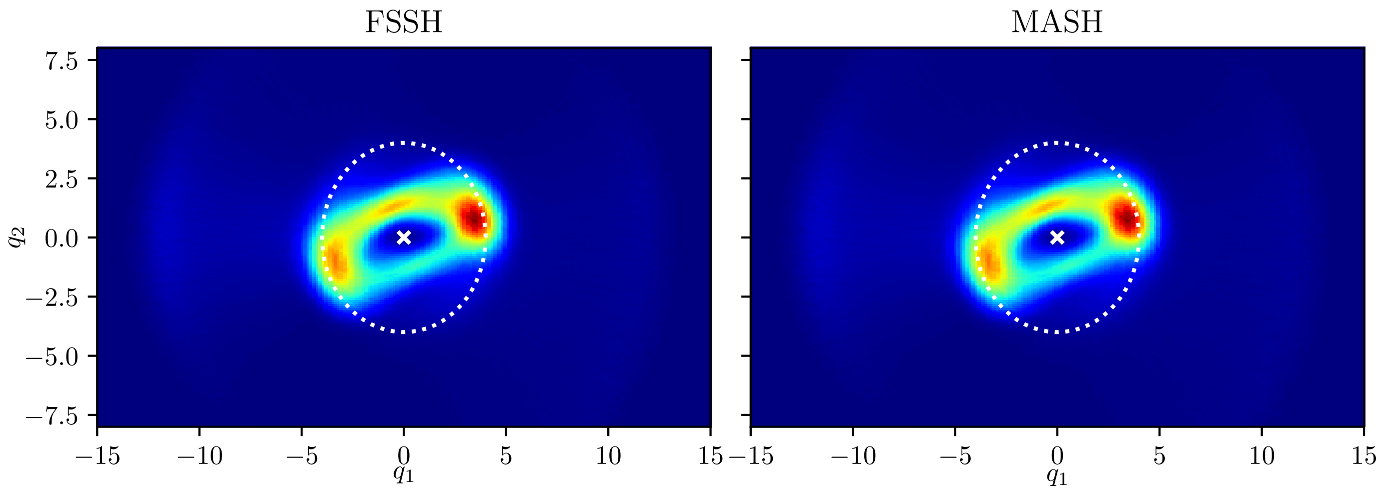

Figure S4: MASH and FSSH nuclear density averaged over the simulation time, both indistinguishable from the QM result given in Fig. 3 of the main text.

Model of acrolein The parameterized model was obtained from Ref. 7, with some slight modifications. In particular, we added fourth-order terms in q to the diagonal elements of the diabatic Hamiltonian, in order to ensure that the potential is bounded from below. This did not significantly change the potential in the region of interest, but was necessary since classical trajectories that escape to the unbounded region will diverge. This issue was not previously encountered in the full QM description due to the finite size of the grid. In the present work we use same the fourth-order diabatic Hamiltonian for all methods, and note that the full QM description leads only to slight differences with the previous work. The diabatic Hamiltonian is defined as:

$$\hat{V} = \begin{pmatrix} H_{\mathrm{AA}}(q_1, q_2) & f_{\mathrm{AB}}(q_1, q_2) g(q_1) \mathrm{e}^{-q_2^2/0.08} \\ f_{\mathrm{AB}}(q_1, q_2) g(q_1) \mathrm{e}^{-q_2^2/0.08} & H_{\mathrm{AB}}(q_1, q_2) \end{pmatrix} \tag{5}$$

where

$$g(q_1) = \begin{cases} \mathrm{e}^{-q_1^2/0.18} & q_1 < 0 \\ \mathrm{e}^{-q_1^2/0.045} & q_1 \geq 0 \end{cases}$$

$$\begin{aligned} H_{\mathrm{AA}}, f_{\mathrm{AB}}, H_{\mathrm{BB}} = {} & c_{00} + c_{10}q_1 + c_{01}q_2 + c_{20}q_1^2 + c_{11}q_1q_2 + c_{02}q_2^2 + c_{30}q_1^3 + c_{21}q_1^2q_2 + \\ & + c_{12}q_1q_2^2 + c_{03}q_2^3 + c_{40}q_1^4 + c_{04}q_2^4 \end{aligned}$$

and all coefficients are reported in Table S4. Figure S5 shows the diabatic energies (H_{AA}, H_{AB}), diabatic coupling H_{BB}, adiabatic energies (V_0, V_1) and nonadiabatic coupling $d = \langle\phi_0|\nabla|\phi_1\rangle$. The dashed black lines indicate where the diabatic energies are equal, and the solid black lines indicate where the diabatic coupling is zero. Therefore the CIs are located at the two points where the

black lines intersect. The mass is $m = 30\,000$ a.u., The initial nuclear wavepacket is a Gaussian on

Table S4: Constants for the model of acrolein in atomic units.

	c_{00}	c_{10}	c_{01}	c_{20}	c_{11}	c_{02}
H_{AA}	−0.01854	−0.02817	−0.114	0.3156	−0.1576	0.2457
f_{AB}	0.0006653	−0.05699	−0.001481	−0.02017	−0.06204	0.02157
H_{BB}	−0.001247	0.01804	0.02297	0.4546	−0.2419	0.2242
	c_{30}	c_{21}	c_{12}	c_{03}	c_{40}	c_{04}
H_{AA}	0.1237	0.2883	−0.2856	0.1071	0.08	0.08
f_{AB}	0.06652	0.05527	0.04719	0.02031	0.0	0.0
H_{BB}	0.2404	0.1135	−0.3448	0.07928	0.08	0.08

the excited state centered at $\bar{q} = (0.5, 0.5)$ with a spread corresponding to $\omega = 0.000125$ in both directions.

$$\chi_1(q, t=0) = \sqrt{\frac{m\omega}{\pi\hbar}}\, \mathrm{e}^{-\frac{m\omega}{2\hbar}(q_1-\bar{q}_1)^2}\, \mathrm{e}^{-\frac{m\omega}{2\hbar}(q_2-\bar{q}_2)^2} \tag{6a}$$

$$\rho_1(q_1, q_2, p_1, p_2, t=0) = \frac{1}{\pi^2\hbar^2}\, \mathrm{e}^{-\frac{m\omega}{\hbar}(q_1-\bar{q}_1)^2}\, \mathrm{e}^{-\frac{m\omega}{\hbar}(q_2-\bar{q}_2)^2}\, \mathrm{e}^{-\frac{p_1^2}{m\hbar\omega}}\, \mathrm{e}^{-\frac{p_2^2}{m\hbar\omega}} \tag{6b}$$

The timestep for the simulation is $\delta t = 0.02$ a.u. For MASH and FSSH the convergence has been ensured using 10^5 trajectories, although almost perfect convergence is already obtained with 10^4 trajectories and reasonable results are available from 10^3 trajectories [Fig. S6]. Note that both MASH and FSSH converge at the same rate.

The standard deviations for the Gaussian envelopes of the electric pulses are respectively $\Delta\omega_{\mathrm{B}} = 5$ and $\Delta t_{\mathrm{N}} = 48$ (both in atomic units).

$$\mathcal{E}_{\mathrm{B}}^*(\omega) = \mathrm{e}^{-\frac{\omega^2}{2\Delta\omega_{\mathrm{B}}^2}} \tag{7a}$$

$$\mathcal{E}_{\mathrm{N}}(t) = \mathrm{e}^{-\frac{t^2}{2\Delta t_{\mathrm{N}}^2}} \tag{7b}$$

The Wigner spectrogram, $\mathcal{W}(T, \omega)$, is defined using a reference frequency of $\omega_{\mathrm{R}} = -0.05$.

To provide a simple interpretation of the TRUECARS signal from the various simulations, we employ the following idealizations to obtain the histogram of adiabatic energy gaps:

1. Most of the time, the nuclear density is outside of coupling region, where

$$\alpha_z(q) \approx 0, \qquad \alpha_x(q) \approx 1. \tag{8}$$

Therefore the time-dependent polarizability is given by

$$\langle \alpha(t) \rangle \approx \langle \sigma_x(t) \rangle \tag{9}$$

2. We assume that $\mathcal{E}_{\mathrm{N}}(t)$ is slowly decaying with time with respect to the frequencies $\omega_t = \Delta V(q(t))$ but faster than the timescale of the nuclear dynamics. We thus approximate $\sigma_x(t) =$

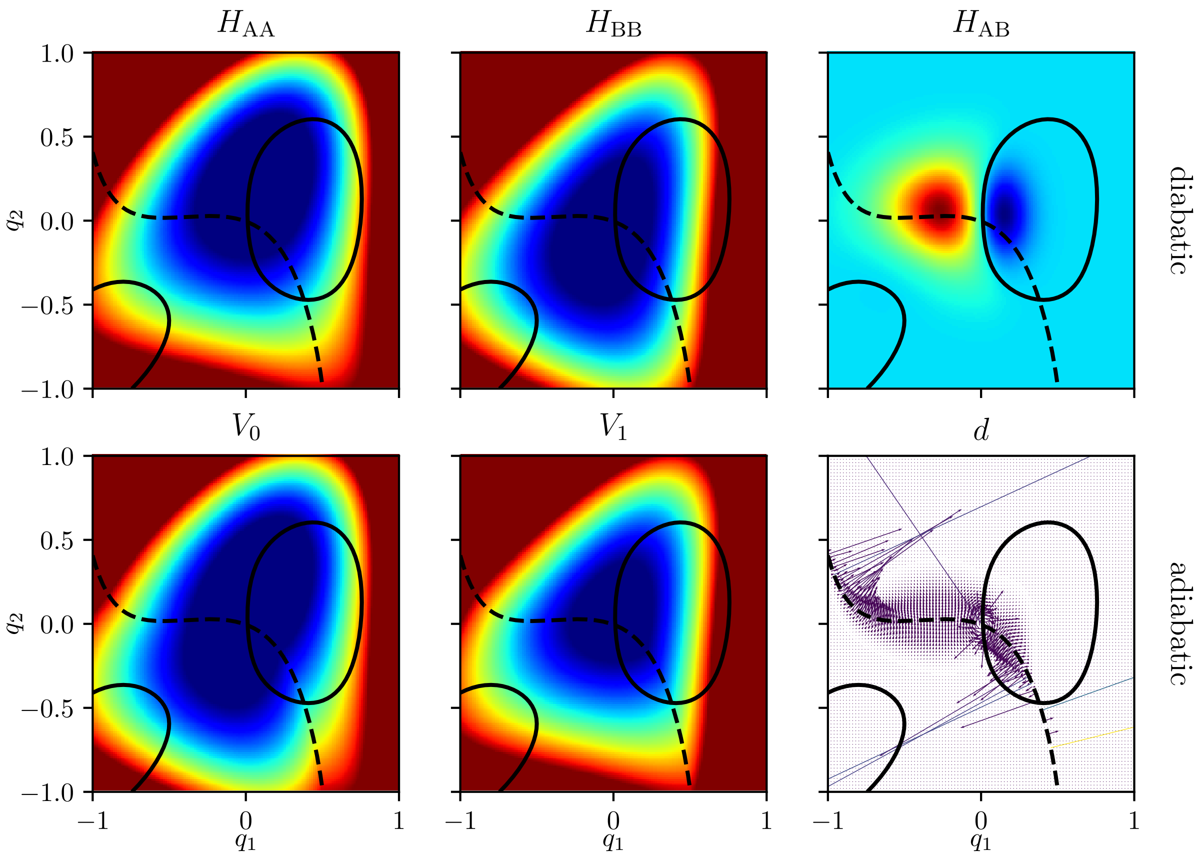

Figure S5: Diabatic and adiabatic energies, diabatic coupling and nonadiabatic coupling vectors, d, for the model of acrolein.

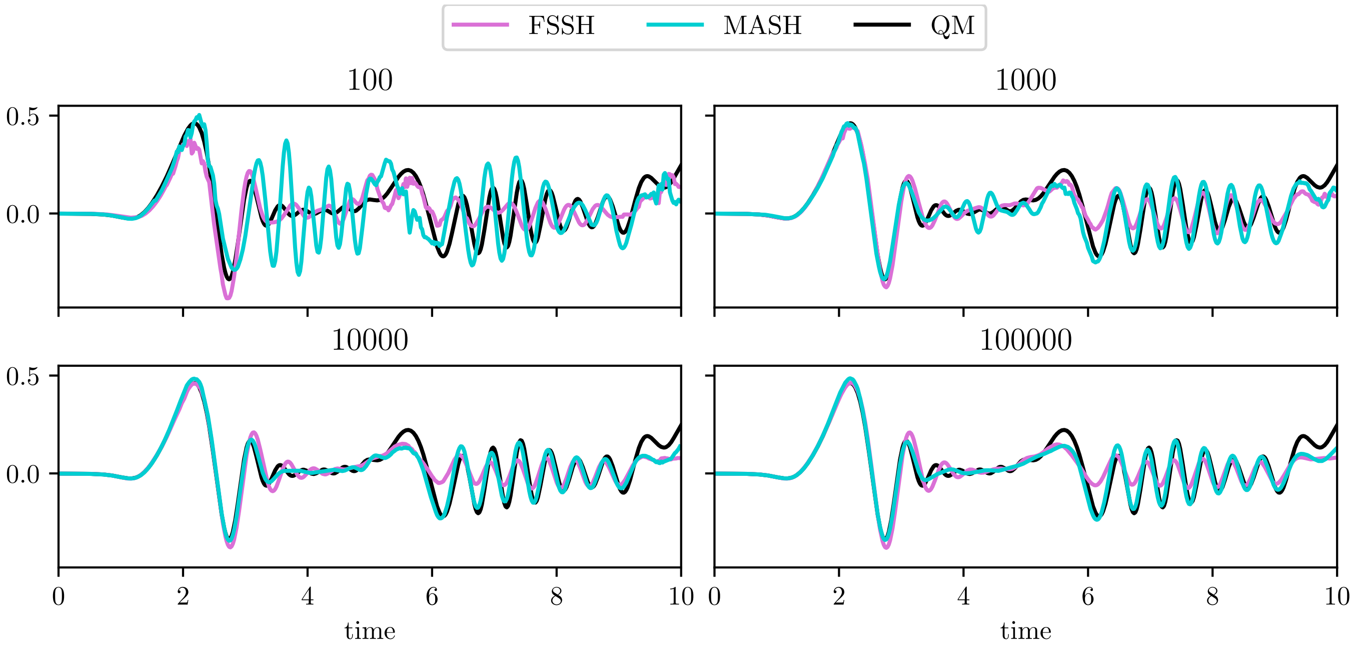

Figure S6: MASH and FSSH polarizabilities averaged over different numbers of trajectories for the model of acrolein.

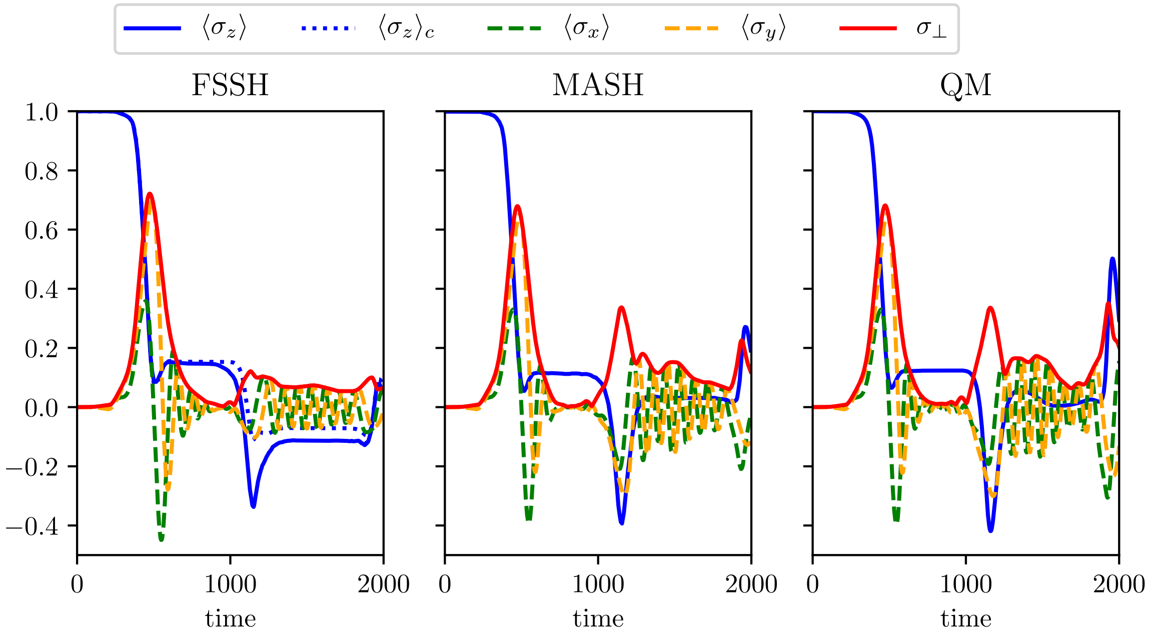

Figure S7: Adiabatic populations and coherences for the model of acrolein. For FSSH, we also plot $\langle\sigma_z(t)\rangle_c$ calculated from the coefficients $\langle|c_1(t)|^2 - |c_0(t)|^2\rangle_{\text{FSSH}}$. We also show the absolute value of the coherences $\sigma_\perp = \sqrt{\langle\sigma_x\rangle^2 + \langle\sigma_y\rangle^2}$ in red.

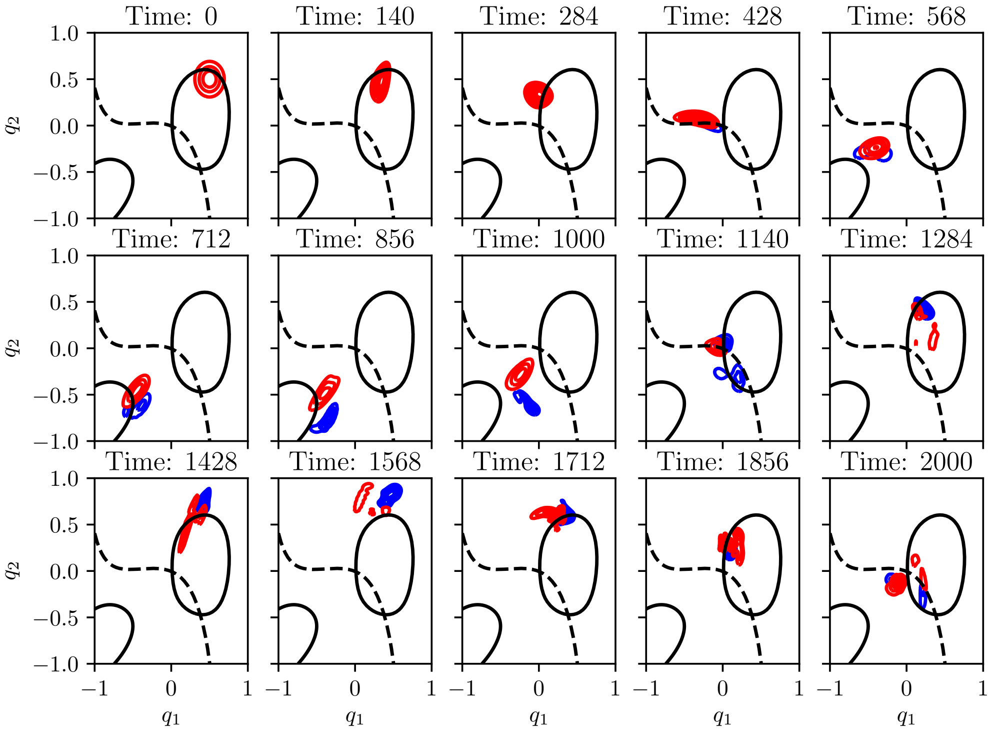

Figure S8: Quantum dynamics of the model of acrolein. The nuclear density on the upper adiabatic state is plotted in red and on the lower adiabatic state in blue.

$\sigma_\perp(T)\sin(\omega_t t)$, where

$$\text{QM} \qquad \langle\sigma_x(t)\rangle = 2\int \mathrm{d}q|\chi_1(q,t)\chi_0(q,t)|\sin(\Delta V(q)t) \tag{10a}$$

$$\text{MASH} \qquad \langle\sigma_x(t)\rangle = 2\left\langle h(S_z(0))\sqrt{S_x(t)^2+S_y(t)^2}\sin\left[\Delta V(q(t))t\right]\right\rangle_{\text{MASH}} \tag{10b}$$

$$\text{FSSH} \qquad \langle\sigma_x(t)\rangle = 2\left\langle|\langle c_0(t)c_1(t)\rangle|\sin\left[\Delta V(q(t))t\right]\right\rangle_{\text{FSSH}} \tag{10c}$$

3. We assume that $\mathcal{E}^*_{\mathrm{B}}(\omega)$ is so broad as to be effectively uniform for all relevant frequencies.

The histogram of the signal is thus reduced to:

$$\begin{aligned}
P(\omega',T) &= \int_0^\infty \mathrm{d}\omega\,\mathcal{S}(\omega,T)\,\delta(\omega-\omega') && (11a)\\
&= \int_0^\infty \mathrm{d}\omega\,2\,\mathrm{Im}\int_{-\infty}^{+\infty}\mathrm{d}t\,\mathrm{e}^{\mathrm{i}\omega(t-T)}\mathcal{E}^*_{\mathrm{B}}(\omega)\mathcal{E}_{\mathrm{N}}(t-T)\langle\sigma_x(t)\rangle\,\delta(\omega-\omega') && (11b)\\
&\approx \int_0^\infty \mathrm{d}\omega\,2\,\mathrm{Im}\int_{-\infty}^{+\infty}\mathrm{d}t\,\mathrm{e}^{\mathrm{i}\omega(t-T)}\mathcal{E}_{\mathrm{N}}(t-T)\langle\sigma_\perp(T)\sin(\omega_t t)\rangle\,\delta(\omega-\omega') && (11c)\\
&\approx \int_0^\infty \mathrm{d}\omega\,2\pi\langle\sigma_\perp(T)\delta(\omega-\omega_t)\rangle\,\delta(\omega-\omega') && (11d)\\
&= 2\pi\langle\sigma_\perp(T)\delta(\omega-\omega')\rangle && (11e)
\end{aligned}$$

Therefore the average shift is defined as

$$\bar{\omega}(t) = \frac{\langle\sigma_\perp(t)\,\omega_t\rangle}{\langle\sigma_\perp(t)\rangle} \tag{12}$$

or more explicitly for each method:

$$\text{QM} \qquad \bar{\omega}(t) = \frac{\int \mathrm{d}q\,|\chi_1(q,t)\chi_0(q,t)|\,\Delta V(q)}{\int \mathrm{d}q\,|\chi_1(q,t)\chi_0(q,t)|} \tag{13a}$$

$$\text{MASH} \qquad \bar{\omega}(t) = \frac{\langle h(S_z(0))\sqrt{S_x(t)^2+S_y(t)^2}\,\Delta V(q(t))\rangle_{\text{MASH}}}{\langle h(S_z(0))\sqrt{S_x(t)^2+S_y(t)^2}\rangle_{\text{MASH}}} \tag{13b}$$

$$\text{FSSH} \qquad \bar{\omega}(t) = \frac{\langle|c_1(t)c_0(t)|\,\Delta V(q(t))\rangle_{\text{FSSH}}}{\langle|c_1(t)c_0(t)|\rangle_{\text{FSSH}}} \tag{13c}$$

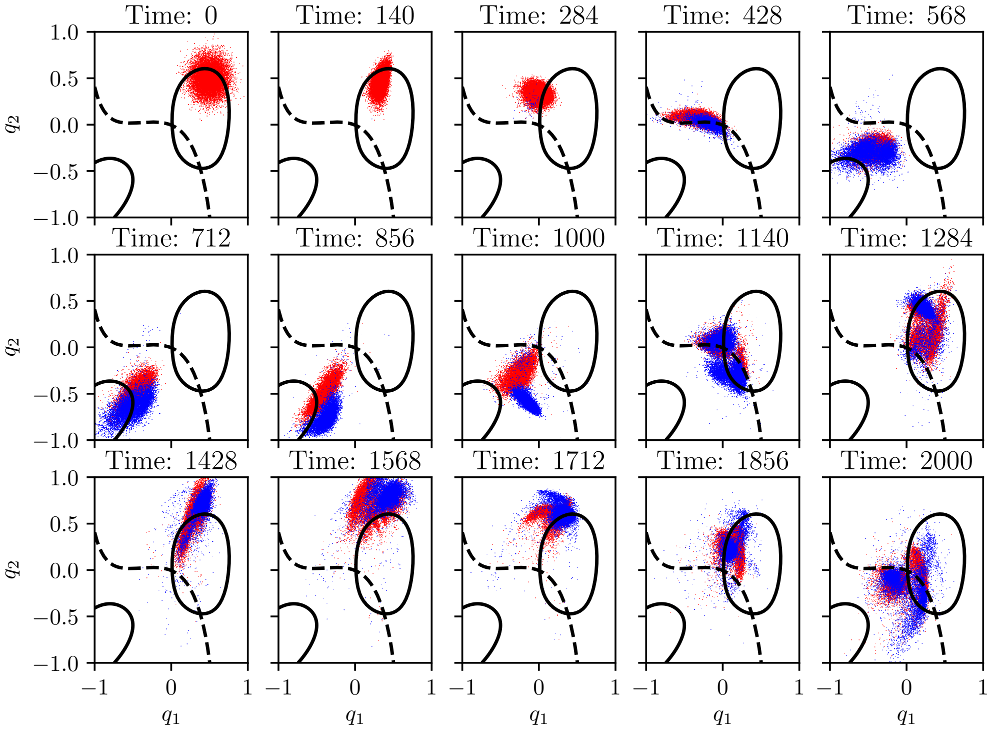

Figure S9: MASH dynamics of the model of acrolein. The trajectories on the upper (lower) adiabatic state are red (blue) points.

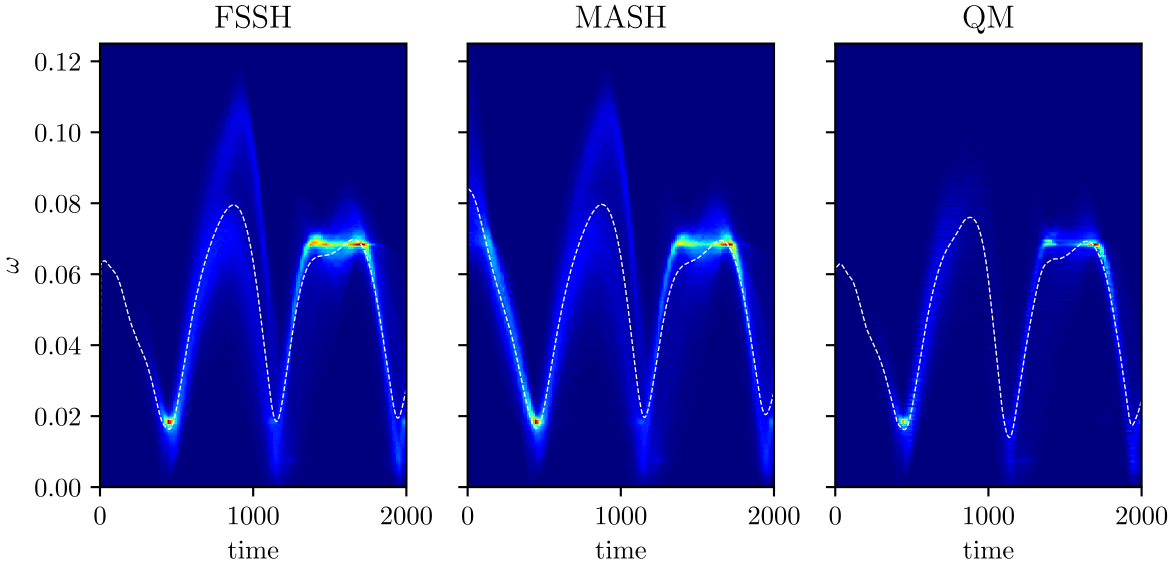

Figure S10: Energy shift between the adiabatic states ΔV, weighted by the corresponding factor in [Eq. (13)]: $|\chi_1(q,t)\chi_0(q,t)|$, $\sqrt{S_x(t)^2 + S_y(t)^2}$ and $|c_1(t)c_0(t)|$. In each case, we also plot the mean frequency $\bar{\omega}(T)$ as a white dashed line. Note that these definitions are not true physical observables; they are just useful to interpret the signal.
