# Supplementary material for: Simulating Electronic Coherences Induced by Conical Intersections Using MASH: Application to Attosecond X‑ray Spectroscopy
Source: J Phys Chem Lett. 2025 Jun 25;16(26):6794–800. doi: 10.1021/acs.jpclett.5c01407 (PMC12235616; doi:10.1021/acs.jpclett.5c01407)
Supplement: Supplementary file 2 [file jz5c01407_si_002.pdf]

Eidgenössische Technische Hochschule Zürich
Swiss Federal Institute of Technology Zurich

Department of Chemistry and Applied Biosciences
Director of Studies for Interdisciplinary Sciences

ETH Zurich
Prof. Dr. Jeremy O. Richardson
Theoretical Molecular Quantum Dynamics
HCI D 267.3
Vladimir-Prelog-Weg 2
8093 Zurich, Switzerland

Phone +41 44 633 46 36
jeremy.richardson@phys.chem.ethz.ch

Editors
The Journal of Physical Chemistry Letters

Zurich, 12th June 2025

Manuscript Submission

Dear Prof. Prezhdo,

Please find enclosed the revised manuscript entitled “Simulating electronic coherences induced by conical intersections using MASH: Application to attosecond X-ray spectroscopy.” We have made minor revisions according to the suggestions of the reviewers.

In particular, following the first suggestion of Reviewer 1, we added a paragraph and Figure S2 in the SI, illustrating the convergence properties of MASH and FSSH. From these results, it is clear that populations and coherences converge with the same rate, proportional to 1/sqrt(N), as expected. However, the prefactor is different for the two methods, with a slightly slower convergence for the coherences in MASH. However, importantly, MASH converges to the correct result, whereas FSSH has a systematic error. The convergence of the populations shows no significant difference between the two methods.

Reviewer 1 also asked for more information on the derivation of the TRUECARS signal. Instead of repeating the work presented in Ref. 7, we simply provide an extra citation to refer the reader to the original work.

Following the suggestion of Reviewer 2, we mention the presence and the position of nodes in the nuclear density in the caption of Figure 2.

I hope that you will now find our manuscript acceptable for publication.

Yours sincerely

Jeremy Richardson

Jeremy Richardson
